# Brugada Syndrome: Anesthetic Implications in an Incidentally Diagnosed Patient

**DOI:** 10.7759/cureus.66963

**Published:** 2024-08-15

**Authors:** Catia Domingues, Luís Gonçalves, Rita Alves, Andreia Mafra, Lucia Gonçalves

**Affiliations:** 1 Anesthesiology, Unidade Local de Saúde da Região de Leiria, Leiria, PRT

**Keywords:** regional anesthesia, combined anesthesia, perioperative management, ventricular arrhythmias, brugada syndrome

## Abstract

Brugada syndrome (BS) is characterized by abnormal repolarization in cardiac cells, occurring in the absence of structural heart disease, which elevates the risk of ventricular arrhythmias and sudden cardiac death. While most BS patients are asymptomatic, a notable percentage experience syncope or sudden cardiac death. Diagnosis is primarily based on electrocardiographic (ECG) findings. A 40-year-old male with a history of syncope and a family history of sudden cardiac death was scheduled for urgent clavicle osteosynthesis. Preoperative ECG revealed type 1 BS. A multidisciplinary approach was taken, and anesthetic management involved combined general and regional anesthesia, utilizing ultrasound-guided clavipectoral and superficial cervical blocks. Postoperative pain was managed with paracetamol and ketorolac. The patient remained stable throughout the procedure, was monitored for 36 hours postoperatively, and was discharged without complications. BS poses significant perioperative risks, necessitating careful anesthetic management. This case report highlights the successful use of combined general and regional anesthesia in a BS patient, contributing to the limited evidence on safe anesthesia practices for this pathology.

## Introduction

Brugada syndrome (BS) is characterized by repolarization abnormalities in cardiac cells in the absence of structural heart disease. These changes in transmembrane ion channels increase the susceptibility to arrhythmias, increasing the risk of sudden cardiac death [1,2]. The estimated prevalence of BS is five cases per 10,000 individuals, being 8-10 times more frequent in men as compared to women. This syndrome is more prevalent in Asia, where it is thought to be endemic, and where it is the most important cause of sudden death in young males [1,2].

Autosomal dominant and sporadic mutations may contribute to sodium and calcium channel abnormalities leading to an imbalance between the inflow and outflow currents during the cardiac action potential. This can create a vulnerable window during which an extrasystole may trigger a re-entry arrhythmia, increasing the risk of ventricular arrhythmias. Repolarization abnormalities can be augmented by several factors, such as changes in the autonomic tone, temperature, antiarrhythmic agents, anesthetic agents, psychotropic drugs, cocaine, and alcohol [3-5].

While most BS patients are asymptomatic, 17-42% may present with sudden cardiac death secondary to ventricular arrhythmia. Symptoms can manifest at any age, but the onset is usually around the fourth decade. Prodromal symptoms, which may precede fatal events, include syncope and paroxysmal palpitations. Supraventricular arrhythmias (most commonly atrial fibrillation) may also affect up to 20% of patients. These symptoms usually occur when vagal activity predominates (at rest or sleep) [1,5].

Diagnosis of BS is primarily based on electrocardiographic (ECG) findings, with type 1 abnormality being the only definitive diagnostic criterion. This is characterized by a coved ST-segment elevation >0.2 mV in more than one right precordial lead (V1-V3), followed by a negative T wave [4,5]. This ECG pattern can be intermittent, requiring multiple tests to detect the abnormality. For patients with a suspected diagnosis but without confirmatory ECG findings, a pharmacological challenge using a sodium channel-blocking drug may be performed under continuous monitoring [6].

Other abnormality types include type 2 (saddle-like ST elevation with >0.2 mV in right precordial leads followed by positive or isophasic T waves) and type 3 (either type 1 or type 2, but with <0.1 mV of ST-segment elevation) [4, 5]. Differential diagnosis with similar ECG patterns (eg., acute myocardial infarction, pericarditis, atypical right bundle branch block, pulmonary embolism, early repolarization syndrome, and electrolyte disorders) should be done [5,7].

Long-term management of patients with BS remains challenging, with treatment options primarily limited to implantable cardioverter defibrillator (ICD) placement, quinidine, and cilostazol. In acute settings, isoprenaline infusion may also be an effective option [1,5,6]. During the anesthetic-surgical procedure, the physiological changes that occur, combined with drug administration, predispose patients with BS to an increased risk of ventricular arrhythmias. Therefore, it is crucial to have a comprehensive perioperative management plan for these patients [5,7]. Preoperatively, a thorough history should be taken, including an inquiry about symptoms, previous cardiac events, and the presence of an ICD. A 12-lead ECG may help stratify risk, and electrolyte levels should be checked and corrected if necessary. A multidisciplinary approach, involving the perioperative team and a cardiologist, is essential to ensure adequate optimization [5].

Induction agents such as benzodiazepines, barbiturates, etomidate, and propofol have all been used successfully [2]. Adverse events related to propofol occur mainly during prolonged infusions at high rates. Ketamine and etomidate have been associated with ST-segment elevation in some cases [5]. Inhaled anesthetics are considered a safe option and sevoflurane is the drug of choice in this class [4]. Depth of anesthesia monitoring should be used to limit general anesthetic doses [1,7]. No adverse effects have been reported with the use of non-depolarizing blocking agents. It is suggested to avoid neostigmine in these patients; however, if necessary, an anti-muscarinic should be administered beforehand [3,4,7]. Opioids can be used safely, with those of short action being preferred [5].

Regional anesthesia is not contraindicated in BS, but local anesthetics should be administered with caution in areas where systemic absorption may be rapid [1,4]. Most adverse events have occurred in cases where BS had not been previously diagnosed [1]. Although there is conflicting evidence for the use of regional anesthesia, it presents some advantages such as providing better analgesia and, in some cases, avoiding general anesthesia and its associated autonomic changes. Local anesthetics with slow sodium channel dissociation in the cardiomyocytes, such as bupivacaine, should be avoided, as they can increase the risk of ventricular arrhythmias. Short-acting anesthetics should be considered the first option for these patients [4,7,8]. Ultrasound and aspiration before injection are simple techniques that can prevent serious adverse effects related to the systemic absorption of local anesthetics.

To minimize the risk of triggering arrhythmic events in patients with BS undergoing surgery, the anesthesiologist must carefully consider pharmacological and monitoring strategies when establishing anesthetic and analgesic plans. This case report discusses the anesthetic and analgesic management of a patient with an incidental diagnosis of BS, who had to undergo urgent osteosynthesis of the clavicle.

## Case presentation

**Figure 1:**
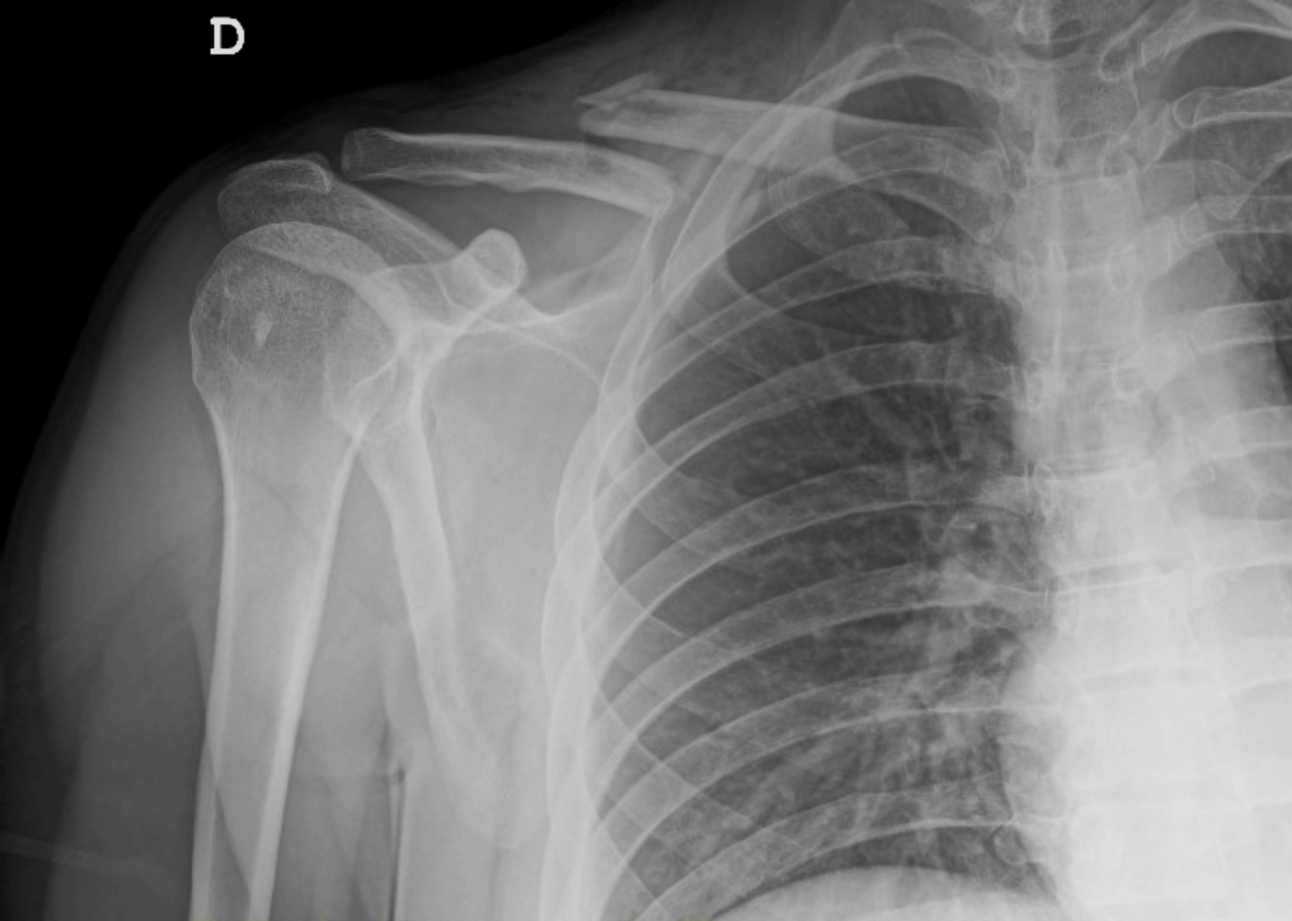
Preoperative X-ray image of right clavicle fracture resulting from trauma

A 40-year-old man, American Society of Anesthesiologists (ASA) II, was scheduled for urgent osteosynthesis of the right clavicle fracture resulting from trauma (Figure 1).

Relevant medical history included two syncopal episodes in the previous five years occurring in the context of intense pain and attributed to vasovagal activity. Family medical history was notable for the sudden cardiac death of his grandfather during sleep at the age of 60.

**Figure 2:**
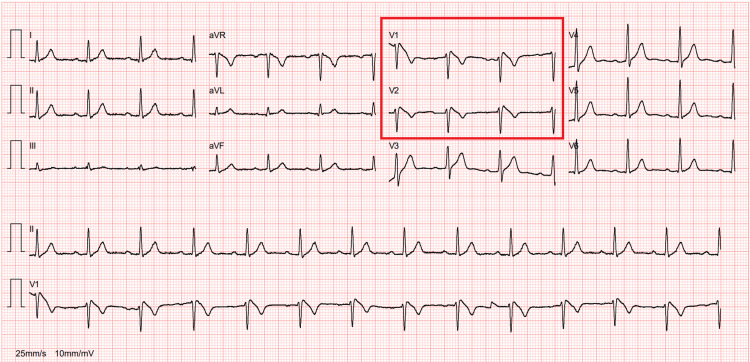
Brugada syndrome type 1 abnormality (coved ST segment elevation > 0.2 mV in V1 and V2 leads, followed by a negative T wave)

All the preoperative diagnostic tests were unremarkable, except for the ECG, which showed a coved ST segment elevation >0.2 mV in V1 and V2 leads, followed by a negative T wave (Figure 2). These findings were discussed with the cardiology team, leading to a diagnosis of BS. A multidisciplinary perioperative plan was subsequently developed. The patient was informed of the risks associated with the new diagnosis, as well as the anesthetic and surgical interventions, and provided his informed consent to proceed.

**Figure 3:**
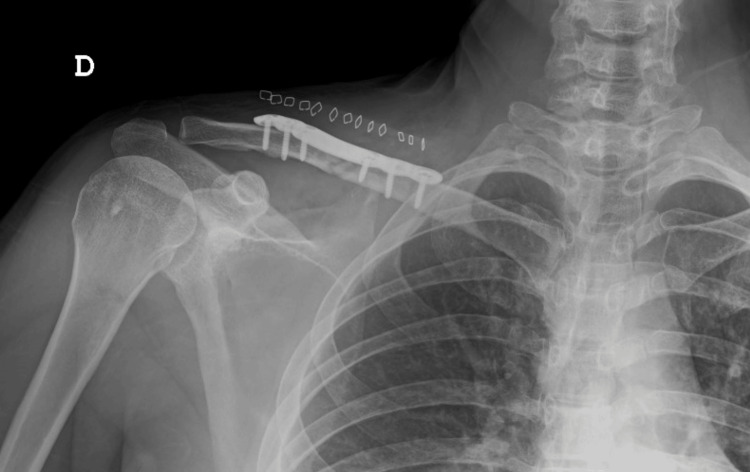
Postoperative X-ray image of right clavicle plate osteosynthesis

In the operating room, standard ASA monitoring and invasive arterial pressure monitoring were initiated, and external defibrillator pads were placed. Mild sedation was achieved with 2 mg of intravenous midazolam, and ultrasound-guided right clavipectoral and superficial cervical blocks were performed (a total of 20 mL of 0.5% ropivacaine was administered). General anesthesia was induced with intravenous fentanyl 0.15 mg, thiopental 450 mg, and rocuronium 50 mg, and maintained with sevoflurane (guided by depth of anesthesia monitoring). Open clavicle reduction and plate osteosynthesis were performed through an anterior approach, with an estimated blood loss of 20 mL (Figure 3). An intraoperative multimodal approach to pain management included intravenous dexamethasone 8 mg, paracetamol 1 g, and ketorolac 30 mg. Neuromuscular blockade was reversed with 200 mg of sugammadex, confirmed by neuromuscular monitoring using the train-of-four ratio. The patient remained hemodynamically stable throughout the 90-minute surgery and after anesthesia emergence. Postoperatively, the patient was continuously monitored for 36 hours in the post-anesthesia care unit (PACU), during which no hemodynamic instability was observed. Pain was effectively managed with scheduled paracetamol and ketorolac, without the need for rescue opioids. He was then transferred to the orthopedic ward and discharged the following day without complications. The patient continues to be monitored by cardiology and was offered ICD placement, which he declined, opting instead for ongoing clinical surveillance.

## Discussion

BS poses a significant challenge for anesthesiologists due to its complex pathophysiology and the associated perioperative risks. This complexity makes the management of elective procedures difficult and even more demanding in urgent situations. Despite the increasing number of scientific publications on anesthesia management of BS, the available evidence is still limited and is primarily confined to case reports and case series. 

In the present case, the diagnosis of BS was an incidental finding, identified only because the patient underwent a preoperative ECG due to his personal history of syncope and family history of sudden cardiac death. This underscores the importance of performing an ECG during the pre-anesthetic evaluation for patients with cardiac risk factors or a history suggestive of cardiac disease. Perioperative monitoring is crucial for the rapid identification and prevention of hemodynamic changes. Standard monitoring is recommended, and a five-lead ECG should be used to accurately detect ST changes, which are associated with an increased risk of arrhythmias [5]. Invasive blood pressure monitoring allows for the early detection of abrupt hemodynamic changes, facilitating prompt correction. Central venous access should be considered for major interventions. External defibrillator pads should be placed on all patients before induction [1,5].

Inadequate analgesia, superficial anesthesia, vagal stimulation (either surgical or pharmacological), and postural changes are associated with autonomic disturbances and should be minimized as they can promote ventricular arrhythmias. Additionally, maintaining normothermia is important to prevent the development of BS-related ECG changes [1, 7]. Our approach involved a combined general and regional anesthesia. Thiopental was selected for anesthesia induction due to the controversy in the literature regarding the use of propofol in patients with BS. While propofol is often considered a drug to be avoided because of its potential arrhythmic effects in BS patients, a recent randomized controlled trial by Flamée et al. demonstrated its apparent safety when used for anesthesia induction [9]. Neuromuscular blockade was reversed with sugammadex to avoid the use of neostigmine.

Published case reports have demonstrated the use of regional and neuraxial local anesthetics for pain management in patients with BS [3,7,8]. Although local anesthetics carry a risk of contributing to sodium channel blockade, potentially precipitating ventricular arrhythmias, none of these cases reported incidents. We chose ropivacaine due to its safer cardiac profile compared to other local anesthetics with a similar duration of action. In this case, the peripheral blockade was not associated with any adverse event and allowed for lower doses of general anesthetics, reducing the risk of cardiovascular instability associated with high doses of these agents. This approach also facilitated better postoperative pain management with reduced opioid use, further enhancing safety and recovery. Thus, this case report adds to the existing evidence regarding the use of regional anesthesia in BS patients.

After surgery, patients with BS should be monitored for at least 36 hours or for a period equivalent to five half-lives of the anesthetic drugs used [5,7]. In this case, this was achieved in the PACU. Overall, this strategy tailored the anesthetic management to address both the specific needs of the patient and the challenges posed by BS, contributing to a safer and more effective outcome.

## Conclusions

BS presents a significant challenge in the perioperative setting due to its potential to cause life-threatening arrhythmias. This case report underscores the importance of a multidisciplinary approach and meticulous planning in the anesthetic management of these patients. Key strategies include the use of combined general and regional anesthesia to minimize autonomic fluctuations and the careful selection of anesthetic agents to avoid exacerbating this condition. Continuous and comprehensive monitoring, both intraoperatively and postoperatively, is crucial to promptly detect and treat any arrhythmic events. Further research and detailed case reports are necessary to develop guidelines and improve the perioperative care of patients with BS.
